# Venous thromboembolism in adolescents and young adults with acute lymphoblastic leukemia treated on a pediatric-inspired regimen

**DOI:** 10.1038/s41408-024-01178-5

**Published:** 2024-10-31

**Authors:** Shai Shimony, Hari S. Raman, Yael Flamand, Julia Keating, Jonathan D. Paolino, Yannis K. Valtis, Andrew E. Place, Lewis B. Silverman, Stephen E. Sallan, Lynda M. Vrooman, Andrew M. Brunner, Donna S. Neuberg, Ilene Galinsky, Jacqueline S. Garcia, Eric S. Winer, Martha Wadleigh, Richard M. Stone, Jean M. Connors, Daniel J. DeAngelo, Marlise R. Luskin

**Affiliations:** 1 Department of Medical Oncology, Dana-Farber Cancer Institute, Boston, MA USA; 2 Department of Data Science, Dana Farber Cancer Institute, Boston, MA USA; 3 Department of Pediatric Oncology, Dana Farber Cancer Institute & Boston Children’s Hospital, Boston, MA USA; 4 Memorial Sloan Kettering Cancer Institute, New York, NY USA; 5 Leukemia Program, Massachusetts General Hospital, Boston, MA USA; 6 Brigham and Women’s Hospital and Harvard Medical School, Boston, MA USA

**Keywords:** Acute lymphocytic leukaemia, Chemotherapy

## Abstract

Asparaginase (ASP)-containing regimens for acute lymphoblastic leukemia (ALL) are associated with venous thromboembolism (VTE). We evaluated the prevalence, risk factors, role of prophylaxis and clinical impact of VTE among adolescents and young adult (AYA) patients (15–50 years) treated on Dana-Farber Cancer Institute (DFCI) ALL protocols. The 1- and 2-year cumulative incidence of VTE were 31.9% (95% CI: 27.0%, 36.9%) and 33.5% (95% CI: 28.5%, 38.5%) respectively, with most events occurring during ASP-based consolidation phase (68.6%). VTE was more frequent in patients with overweight/obese vs. normal BMI (39.2% vs. 29.0%, *p* = 0.048). In a 1-year landmark analysis, the 4-year overall survival was 91.5%, without difference between patients with vs. without VTE (93.8% vs. 90.0%, *p* = 0.93). Relapse and non-relapse mortality rates were also similar. Among patients treated at Dana-Farber/Harvard Cancer Center, cerebral sinus vein thrombosis occurred in 3.6% of patients (8.5% of VTE events) in comparison to pulmonary embolism (32.9%) and deep vein thromboses (58.6%, 24.4% line-associated). In a Cox regression model for VTE free-time, elevated BMI was associated with shorter VTE free-time (HR 1.94 [95% CI 1.13-3.35], *p* = 0.018), while low molecular weight heparin (LMWH) prophylaxis as time-varying covariate was not. In conclusion, we found that VTE was frequent in AYAs treated on DFCI ALL protocols but did not impact survival outcomes. Overweight/obese BMI increased risk for VTE.

## Introduction

Adolescent and young adult (AYA) patients with acute lymphoblastic leukemia (ALL) may be effectively treated with pediatric-inspired chemotherapy regimens which incorporate asparaginase (ASP) [1, 2]. ASP catalyzes the breakdown of asparagine, an amino acid essential for leukemic cell survival, and has a unique set of toxic effects including hypersensitivity, pancreatitis, metabolic derangement and increased risk for venous thromboembolism (VTE) [3, 4]. Risk for VTE is believed to be related to decreased synthesis of anticoagulant proteins including plasminogen, antithrombin, protein C, and protein S [5]. The reported rates of VTE in AYAs with ALL being treated with ASP-containing regimens ranges from 5% to 34% [4, 6–8], with VTE reported most commonly during induction [9].

Prior studies have reported older age and lymphadenopathy as characteristics associated with increased risk for VTE in young patients treated with ASP-containing regimens [6, 8]. Other studies have explored the ability of various pharmacologic and transfusion strategies to reduce risk of thrombotic complication with conflicting results regarding the efficacy of specific prophylaxis strategies [9–11]. Finally, the long-term clinical impact of VTE on survival is not well defined. We conducted this retrospective study to describe the prevalence, risk factors, and clinical impact of VTE among AYA patients treated for ALL on Dana-Farber Cancer Institute (DFCI) ALL Consortium Protocols, and to determine the role of heparin prophylaxis in this clinical context.

## Methods

### Patients

We included consecutive AYAs aged 15–50 years diagnosed with Philadelphia chromosome-negative ALL treated on four multi-center DFCI Consortium protocols (Pediatric 00-001 [12], Pediatric 05-001 [13], Adult 01-175 [1], and Adult 06-254 [14]), or treated as per these protocols between 2000–2021 (Consortium cohort, Supplementary Fig. 1).

All regimens included an ASP-containing induction, 30 weeks of continuous ASP exposure during consolidation and a continuation phase without ASP. The use of either pegylated ASP (peg ASP) or non-pegylated ASP (non-peg ASP) formulations varied between protocols. Notably, no routine VTE prophylaxis was used in the 00-001, 05-001, 01-175 protocols; however, after a high incidence of VTE events was observed in the 06-254 protocol, an amendment was implemented (on September 2011), so that all subsequent patients on 06-254 or treated as per 06-254 regimen received LMWH thromboprophylaxis at standard prophylactic dosing during ASP treatment and antithrombin repletion for activity level <30% of normal. All patients were treated with therapeutic dose anticoagulation once VTE was documented and development of VTE was not considered an indication to stop ASP or be removed from the trial protocol.

Body mass index (BMI) was calculated at day of registration (on trial) or first treatment day (for patients treated as-protocol) and categories classified based on the Center for Disease Control (CDC) guidelines – using age-adjusted percentiles for patients aged <20 years (underweight: <5%; normal: 5–84.99%; overweight: 85–94.99%; obese: ≥95%) and per absolute BMI (kg/m^2^) in patients aged ≥20 years (underweight: <18.5; normal: 18.5–24.99; overweight: 25–29.99; and obese: ≥30). For this analysis, underweight patients (*N* = 10) were classified as normal.

As ASP exposure is associated with both higher VTE rates and better survival, it may confound the association between VTE and survival; thus, to ameliorate confounding, all survival associated outcomes were conducted at a landmark analysis in patients who remained on protocol for at least 1 year (encompassing ASP exposure).

Patients treated at DFCI, Massachusetts General Hospital, Brigham and Women’s Hospital and Boston Children’s Hospital (termed together Dana-Farber/Harvard Cancer Center [DF/HCC] cohort) had detailed data available regarding VTE type, type of access (central vs. peripheral line, type of central line, etc.) and use of prophylaxis. Thus, we conducted a pre-specified subgroup analyses in these patients. Patients with VTE event were considered on prophylaxis if they received any dose of prophylaxis in the 48 h prior to event, which was confirmed through manual chart abstraction. Brief peri-procedural holdings (less than 48 h) of prophylaxis were not considered as stoppage of anticoagulation prophylaxis.

### Outcomes and statistical analyses

Categorical variables are presented as numbers and percentages, and comparisons were made by chi square or Fisher’s exact tests as appropriate. Continuous variables are presented by median and interquartile range (IQR) or range, and comparisons were made by Wilcoxon test. The cumulative incidence of VTE was calculated from date of registration (or first day of treatment for patient treated as-protocol) until VTE event or last follow up by the Fine and Gray method, with death as competing risk. Comparisons between subgroups were made by the Gray test. Univariable and multivariable models were performed to identify associations of VTE with distinct covariates. To integrate the use of prophylaxis as a time-varying covariate into the model in the DF/HCC cohort, we analyzed the cumulative incidence of VTE with cause-specific Cox regression analysis, with death as a censoring event. To evaluate the effect of prophylaxis on VTE incidence within specific subgroups, we conducted Cox regression analyses of prophylaxis within each subgroup separately. Overall survival (OS) was calculated from time of registration (or first day of treatment for patients treated as-protocol) till death or last follow up. Event free survival (EFS) was calculated from time of registration (or first day of treatment for patients treated as-protocol) until progression, death, or last follow-up. Both OS and EFS are presented with Kaplan Meier curves and comparisons are made by the Log-rank test. Cumulative incidence of relapse (CIR) and non-relapse mortality (NRM) were estimated by the cumulative incidence with the Fine and Gray method with the appropriate competing risks and comparisons were made by the Gray test. For all analyses, the confidence interval (CI) was calculated at the (two-sided) 95% confidence level. A two-sided p-value of <0.05 was considered statistically significant. All statistics were performed using SAS software version 9.4, R software version 4.3.2, and Stata software version 17.0.

## Results

### Patients

Overall, 341 AYAs with Philadelphia chromosome-negative ALL were included in the study. The median age was 23.2 years (IQR 17.2–33.3) with the majority male (*n* = 212, 62.2%). With a median follow up of 3.8 years (range 0–16), 114 (33.4%) patients experienced at least one VTE event. Patients with vs. without VTE had higher rates of elevated (overweight/obese) BMI (50.9% vs. 39.6%, *p* = 0.048), lower rates of hyperdiploid karyotype (3.5% vs. 13.2%, *p* = 0.0038) and were more frequently treated on trial (29.8% vs. 20.3% per protocol, *p* = 0.049), Table 1. Of note, hyperdiploid karyotype was also associated with younger age and lower BMI (Supplementary Table 1).

**Table 1:** Dana-Farber Consortium cohort patient characteristics.

	VTE group						
	No (*N* = 227)		Yes (*N* = 114)		Overall (*N* = 341)		*P*-value
	*N*	(%)	*N*	(%)	*N*	(%)	
Sex (Male)	142	(62.6)	70	(61.4)	212	(62.2)	0.84
Age, continuous (median, IQR)	24.3 (17.0, 33.3)		22.3 (17.5, 33.7)		23.2 (17.2, 33.3)		0.79
Age							0.78
15–29 years	156	(68.7)	80	(70.2)	236	(69.2)	
30–50 years	71	(31.3)	34	(29.8)	105	(30.8)	
BMI group (per CDC guidelines)^a^							0.048
Normal/Underweight	137	(60.4)	56	(49.1)	193	(56.6)	
Overweight/Obese	90	(39.6)	58	(50.9)	148	(43.4)	
WBC at diagnosis (x10^9^/L, median, IQR)	16.0 (4.0, 55.0)		14.6 (5.4, 59.0)		15.6 (4.2, 57.0)		>0.99
Immunophenotype							0.18
B-ALL	167	(73.6)	76	(66.7)	243	(71.3)	
T-ALL	60	(26.4)	38	(33.3)	98	(28.7)	
CNS involvement^b^							0.62
CNS-1	180	(79.3)	86	(75.4)	266	(78.0)	
CNS-2	24	(10.6)	17	(14.9)	41	(12.0)	
CNS-3	6	(2.6)	4	(3.5)	10	(2.9)	
Anterior mediastinal mass^c^	45	(19.8)	30	(26.3)	75	(22.0)	0.18
Cytogenetics							
Normal	71	(31.3)	31	(27.2)	102	(29.9)	0.44
Complex	7	(3.1)	3	(2.6)	10	(2.9)	>0.99
Hyperdiploid	30	(13.2)	4	(3.5)	34	(10.0)	0.0038
KMT2A rearrangements (MLL)	20	(8.8)	4	(3.5)	24	(7.0)	0.08
Treatment type (On trial vs. per protocol)	181	(79.7)	80	(70.2)	261	(76.5)	0.049
Asparaginase type^d^ (PEG vs. non-PEG)	119	(52.4)	68	(59.6)	187	(54.8)	0.33
VTE PPx era^e^ (vs. pre-PPx era)	70	(30.8)	36	(31.6)	106	(31.1)	0.89
AlloHSCT	34	(15.0)	12	(10.5)	46	(13.5)	0.26

*VTE* venous thromboembolism, *IQR* interquartile range, *BMI* body mass index, *CDC* Centers for Disease Control, *WBC* white blood cell count, *ALL* acute lymphoblastic leukemia, *CNS* central nervous system, *MLL* mixed-lineage leukemia, *PEG* pegylated, *PPx* prophylaxis, *AlloHSCT* allogeneic hematopoietic stem cell transplantation.

^a^BMI Cutoff: For patients ≥20 years, normal (including underweight): <24.99, overweight/obese: >30. For patients 15-19, age-adjusted percentiles per CDC were used where overweight/obese ≥85%.

^b^17 patients had traumatic tap/unknown CNS status.

^c^Three patients mediastinal mass evaluation was unknown.

^d^Nine patients had unknown type of asparaginase.

^e^At 09/2011, after finding high prevalence of VTE events in the 06-254 protocol, any patients on the 06-254 or treated as per 06-254 received thromboprophylaxis during asparaginase treatment and antithrombin repletion when < 30%.

### VTE characteristics

In the Consortium cohort, VTE events were documented in 140 instances among 114 patients with events most commonly occurring during consolidation (*n* = 96, 69%), followed by induction (*n* = 32, 23%), and uncommonly during continuation/follow-up (*n* = 12, 8%). Among the 114 patients with VTE, 19 (17%) experienced more than one VTE. In total, 10 patients (8.7% of patients with VTE; 2.9% in the overall cohort) experienced cerebral sinus vein thrombosis (CSVT), with one out of the ten experiencing a second CSVT event. Five of the patients (50%) were rechallenged with ASP and there were no additional VTE events, bleeding or evidence of ALL relapse among re-challenged patients. Further details of the 10 patients with CSVT events are summarized in Supplementary Table 2.

### VTE incidence and associated factors

**Fig. 1:**
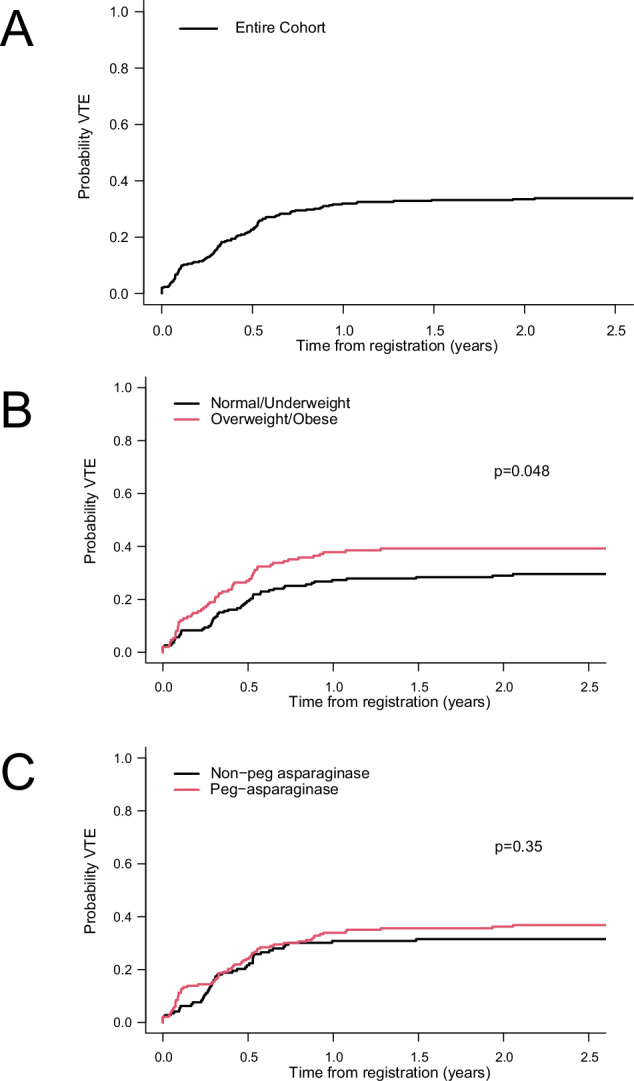
Cumulative incidence of VTE. **A** Entire cohort. **B** Stratified by BMI group (normal vs. overweight/obese). **C** Stratified by type of asparaginase (E. coli vs. peg-asparaginase). VTE venous thromboembolism, BMI body mass index.

The 1-month, 6-months and 1-year and cumulative incidence of VTE were 6.7% (95% CI: 4.4%, 9.7%), 22.7% (95% CI: 18.4%, 27.2%) and 31.9% (95% CI: 27.0%, 36.9%), respectively. The 2-year cumulative incidence of VTE in the Consortium cohort was 33.5% (95% CI: 28.5%, 38.5%, Fig. 1A) and was higher in patients with overweight/obese vs. normal BMI (39.2% [95% CI: 31.3%, 47.0%] vs. 29.0% [95% CI: 22.7%, 35.6%], *p* = 0.048, Fig. 1B). In a competing risk regression model, BMI was the only variable associated with higher VTE risk (HR 1.45 [95% CI: 1.01, 2.09], *p* = 0.047, Supplementary Table 3). Of note, type of ASP (pegylated vs. non-pegylated ASP) did not affect VTE incidence (HR 1.2 [95% CI: 0.82, 1.74], *p* = 0.35, Fig. 1C).

### Effect of VTE on survival

**Fig. 2:**
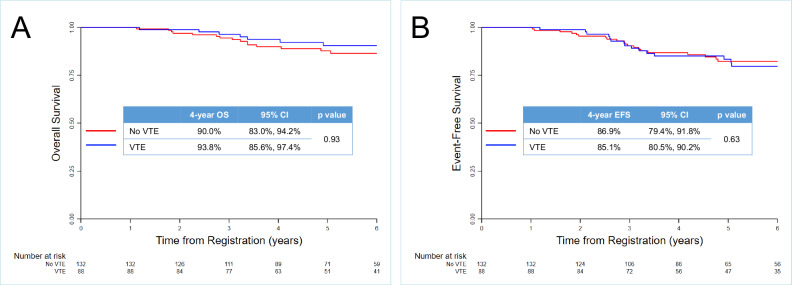
Survival outcomes among AYAs stratified by VTE occurrence. **A** Overall survival. **B** Event free survival. OS overall survival, CI confidence interval, VTE venous thromboembolism, EFS event free survival.

Among patients who completed at least 1 year of protocol therapy (*n* = 220), the 4-year OS was 91.5% [95% CI: 86.7%, 94.6%], and did not differ between patients with vs. without VTE (93.8% [95% CI: 85.6%, 97.4%] vs. 90.0% [95% CI: 83.0%, 94.2%] respectively, *p* = 0.93, Fig. 2A). Similarly, there were no differences in 4-year EFS (85.1% [95% CI: 75.2%, 91.3%] vs. 86.9% [95% CI: 79.4%, 91.8%], *p* = 0.63, Fig. 2B), CIR (19.8% [95% CI: 12.2%, 28.8%] vs. 22.6% [95% CI: 16.6%, 29.2%], *p* = 0.2, Supplementary Fig. 2) and NRM (2.7% [95% CI: 0.7%, 7.0%] vs. 6.9% [95% CI: 3.6%, 11.7%], *p* = 0.59, Supplementary Fig. 3). In univariate analysis for OS, obese BMI (≥30 vs. <30 kg/m^2^), higher WBC (as a continuous variable), B-cell immunophenotype, and fewer weeks on ASP therapy were associated with worse OS and included in the multivariable model. All of these covariates except weeks of ASP as a continuous variable remained significant (Table 2). In the uni- and multivariable analyses for EFS, higher WBC, and B-cell immunophenotype were associated with worse EFS (Supplementary Table 4). Notably, VTE was not associated with worse OS or EFS in all regression analyses.

**Table 2:** Univariate and multivariable OS Cox regression landmark analysis for patients on treatment for at least 1 year.

	Univariate HR [95% CI]	*p*-value	Multivariable HR [95% CI]	*p*-value
Thrombosis (as time-varying covariate)	0.97 [0.44–2.17]	0.95	1.22 [0.53–2.79]	0.64
Age as continuous variable (years)	1.02 [0.98–1.06]	0.38	-	-
Sex (Male *vs*. Female)	1.09 [.48–2.46]	0.84	-	-
BMI (overweight/obese *vs*. normal/underweight)	1.59 [0.72–3.48]	0.25	-	-
BMI				
- Overweight *vs*. Normal/Underweight	0.84 [0.27–2.61]	0.77	-	-
- Obese *vs*. Normal/Underweight	2.63 [1.10–6.26]	0.029	-	-
- Obese BMI (Obese *vs*. Underweight/ Normal/Overweight BMI)^a^	2.75 [1.21–6.24]	0.02	2.64 [1.16–6.01]	0.02
WBC at diagnosis	1.003 [1.001–1.004]	<0.01	1.003 [1.001–1.004]	<0.01
CNS-3 *vs*. CNS-1 or CNS-2	1.94 [0.26–14.36]	0.52	-	-
Immunophenotype B- *vs*. T-ALL	5.09 [1.20–21.60]	0.03	4.74 [1.10–20.39]	0.04
Hyperdiploid (yes *vs*. no)	1.21 [.41–3.52]	0.73	-	-
MLL rearrangement (yes *vs*. no)	1.29 [0.17–9.53]	0.81	-	-
Weeks of Asparaginase	0.96 [0.93–1.00]	0.062	0.97 [0.93–1.00]	0.08

*HR* hazard ratio, *CI* confidence interval, *BMI* body mass index, *WBC* white blood cells, *CNS* central nervous system, *ALL* acute leukemia lymphoma, *MLL* mixed lineage leukemia, *BMI* body mass index.

^a^Dichotomous obese variable (obese BMI vs. underweight/normal/overweight BMI) included in multivariable model over other BMI variables, given higher significance.

### Subgroup analysis at DF/HCC cohort

**Fig. 3:**
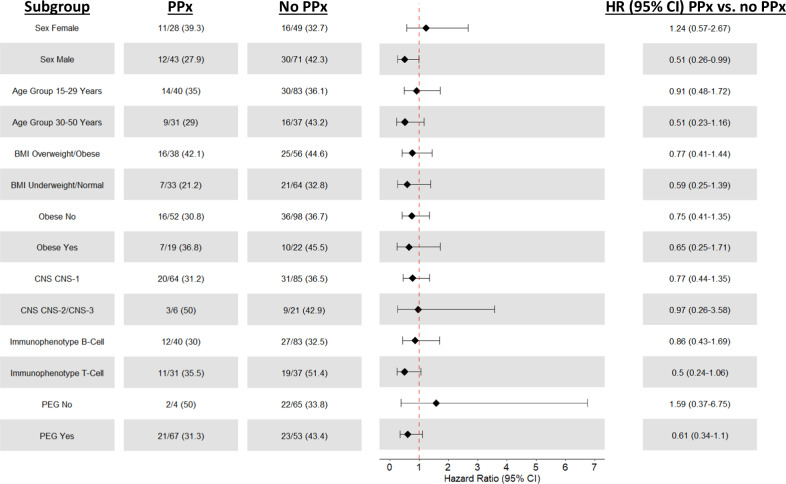
Impact of LMWH on VTE rates in specific subgroups among DF/HCC cohort (*n* = 191). PPx prophylaxis, HR hazard ratio, CI confidence interval, BMI body mass index, CNS central nervous system, PEG pegylated.

In the subgroup treated at DF/HCC (*n* = 191), 69 patients (36.1%) experienced at least one VTE (total events 82, with 11 experiencing more than one event); those with VTE had a higher rate of elevated BMI (59.4% vs. 43.4%, respectively, *p* = 0.034), Supplementary Table 5. VTE prophylaxis was given in 71/191 patients (37%), primarily with fixed-dose low molecular weight heparin (LMWH; 69/71, 97%); one person was treated with apixaban and one with fondaparinux. Similar to the Consortium cohort, most events (*n* = 50, 61%) occurred during consolidation, followed by induction (*n* = 25, 30%; five patients prior to ASP treatment) and infrequently during continuation/follow-up (*n* = 7, 9%). With regard to type of VTE, pulmonary embolism (PE) was documented in 27 cases (32.9% of VTE events), CSVT in 7 (8.5% of VTE events) and line-associated VTE in 20 (24.4% of VTE events, Table 3). The 1-month, 6-months and 1-year cumulative incidence of VTE in this cohort were 9.9% (95% CI: 6.2%, 14.7%), 24.2% (95% CI: 18.3%, 30.5%) and 33.8% (95% CI: 27.1%, 40.6%), respectively. The 2-year cumulative incidence of VTE was 36.6% (95% CI: 29.7%, 43.5%, Supplementary Fig. 4A). Like the Consortium cohort, overweight/obese BMI vs. normal BMI was associated with higher cumulative risk of VTE (43.6% [95% CI: 33.4%, 53.4%] vs. 29.6% [95% CI: 20.7%, 39.0%], *p* = 0.038, Supplementary Fig. 4B). In a Cox univariate regression model for VTE free-time with LMWH prophylaxis as time-varying covariate, both BMI (overweight/obese vs. normal) and immunophenotype (T-ALL vs. B-ALL) were associated with shorter VTE free-time (HR 1.94 [95% CI 1.125-3.35], *p* = 0.018 and HR 1.62 [95% CI 1.005-2.61], *p* = 0.048) (Supplementary Table 6). In the multivariable model, only overweight/obese BMI remained significant. Of note, prophylaxis as time-varying covariate did not affect VTE free-time (HR 0.98 [95% CI: 0.56–1.72], *p* = 0.96). To determine if any subgroup benefited from prophylaxis, we conducted separate regression model for distinct subgroups in an exploratory analysis. The use of prophylaxis was associated with lower risk of VTE in males (HR 0.51 [95% CI 0.26, 0.997], *p* = 0.049) but not in females (HR 1.24 [95% CI 0.57, 2.67], *p* = 0.59), Fig. 3. Furthermore, patients with T-ALL and those who were treated with peg ASP who received prophylaxis had lower rates of VTE, although with marginal statistical significance (HR 0.5 [0.24, 1.06], *p* = 0.07 and 0.61 [95% CI 0.31, 1.1], *p* = 0.1, respectively). Of note, there was no VTE-reducing benefit of prophylaxis in any of the BMI subgroups.

**Table 3:** VTE events per phase and type in the DF/HCC cohort (*n* = 191).

	DF/HCC Cohort		
	Patients evaluated	Patients with VTE (*N*,%)	Number of Events (*N*)^a^
Overall	191	69 (36.1)	82
Induction	191	24 (12.6)	25
Consolidation	170	44 (25.9)	50
Continuation/ Follow-Up	136	7 (5.2)	7
CSVT	69	7 (10.1)	8
PE	69	23 (33.3)	27
Line associated	69	18 (26.1)	20

*VTE* venous thromboembolism, *CSVT* cerebral sinus vein thrombosis.

^a^11 patients had multiple events: 10 patients had two events, and 1 patient had 4 events.

## Discussion

Venous thromboembolism is common among AYAs with ALL treated with ASP-containing regimens [15]. The rate of VTE, the impact of VTE on outcomes and the role of VTE prophylaxis remains unclear and may vary between ALL regimens [1, 2, 8, 16, 17]. We found that in AYAs treated on DFCI consortium protocols VTE events were common (2-year cumulative incidence of 33.5%), occurred most during ASP-based consolidation phase (69%) and were higher in patients with overweight/obese BMI vs. patients with normal BMI (39.2% vs. 29.0%, *p* = 0.048). Notably, VTE incidence did not impact key study outcomes including CIR, NRM, EFS or OS, suggesting that VTE is a manageable toxicity.

The rate of VTE seen in our study is higher compared with previous pediatric inspired regimens in AYAs; for instance, the rate of VTE in the CALGB 10403 and in GRAALL 2005 were ~15% and 16% respectively [7, 9, 15, 18]. Furthermore, most VTE in our cohort occurred during consolidation phase (69%); this is in contrary to previous studies which showed the highest rates during induction phase with lower rates during post-induction phases. This is driven mainly due to the unique continuous ASP exposure for 30 weeks during consolidation phase in the DFCI protocols [1, 12–14] vs. intermittent dosing schedule in other protocols [2].

Our finding of an association between obesity and VTE are additive to our previous findings showing association between obesity and specific toxicities, such as hyperglycemia and elevated liver enzymes, in patients treated on DFCI ASP-containing pediatric regimens [19]. Overall, these findings demonstrate the significant correlation between obesity and toxicity in ASP-containing regimens in ALL and may warrant strict monitoring and management of AYAs with obesity. Of note, a previous study from our group demonstrated the association with older age and risk for VTE [6]. However, the earlier study was mainly composed of children younger than 18 years (91%), whereas our larger study included patients between 15–50 years and no younger children. It may be that age is a significant risk factor for VTE in children between treated with ASP whereas BMI is the factor most associated with increased risk of VTE in adolescents and young adults aged 15–50 years.

We also found that a third of the VTE events were PE, a quarter were line-associated, and only a few experienced CSVTs, most of which were rechallenged without neurological sequelae. Despite the high rates of VTE in our cohort, overall outcomes were excellent and did not differ between patients with vs. without VTE. As multiple studies now show the importance of ASP treatment completion for better survival [20, 21], our results support the DFCI practice of continuing ASP treatment once the treatment of the acute thrombotic complication stabilizes.

In our analysis, we did not demonstrate a benefit of VTE prophylaxis with LMWH in the entire cohort. This was a disappointing finding particularly given the need for effective VTE prevention in this population and highlights the importance of continuing to pursue more effective methods of VTE prophylaxis in the era of novel anti-coagulation therapies. However, we found a benefit in specific subgroups: in males and possibly in T-ALL and those treated with peg-ASP-containing regimens. Results are conflicting in the literature regarding the benefit of thromboprophylaxis in patients with ALL treated with ASP-containing regimens. In the THROMBOTECT trial, both prophylactic antithrombin (AT) repletion and use of LMWH were shown to reduce the incidence of VTE in children and adolescents up to the age of 18 years [22]. Conversely, in the recently published PREVAPIX-ALL trial in children, no benefit was found with apixaban vs. placebo for VTE prophylaxis [23]. No prospective trials evaluating the role of VTE prophylaxis have been conducted in adults. A post hoc analysis of the GRAALL 2005 trial demonstrated that neither AT nor heparin prevented VTE, and that fibrinogen repletion increased the rate of VTE [9]. Conversely, a Canadian study in 225 adult patients demonstrated that LMWH reduced VTE risk vs. no prophylaxis [10]. Overall, the use of prophylactic anticoagulation in ASP-containing AYA protocols remains controversial. Our findings on specific benefit in certain subgroups may guide the use of prophylaxis in certain clinical scenarios (i.e., a male with T-ALL) and may prompt discontinuation of prophylaxis where the net clinical risk-benefit is not clear. Unfortunately, although overweight/obese patients experienced higher VTE rates, the use of LMWH prophylaxis did not seem to mitigate the risk in this population. It may be, in part, due to subtherapeutic doses in obese patients when a fixed prophylaxis dose of LMWH is used [24]. Either dose adjustments and/or alternative approaches should be investigated in obese AYA patients.

Our study has several limitations. First, its retrospective nature may lead to recall bias, although data was centrally collected and verified. Second, we do not have AT measurements and repletion data, thus no clear conclusions can be made on AT repletion as prophylactic management; this may potentially diminish the effect of LMWH prophylaxis. Finally, data on bleeding were lacking, and thus a risk-benefit analysis of bleeding vs. thrombosis could not be done, although a previous study from our group showed the safety of LMWH use in AYAs treated on DFCI consortium protocols [11].

In summary, we found that AYA patients treated with ASP experienced frequent complication of VTE, but this toxicity remains “manageable” with therapeutic anticoagulation, without any impact on curative outcomes. Elevated (overnight/obese) BMI was a risk factor for VTE and the use of prophylaxis with LMWH broadly did not reduce the frequency of VTE or improved survival. Further efforts are needed to mitigate this risk including development of better and more patient friendly pharmacologic and non-pharmacologic risk reduction strategies with emphasis on patients with elevated BMI.

## Supplementary information

Supplementary data

## Data Availability

The data that support the findings of this study are available on request from the corresponding author. The data is not publicly available due to privacy or ethical restrictions.
