# Supplementary material for: Venous thromboembolism in adolescents and young adults with acute lymphoblastic leukemia treated on a pediatric-inspired regimen
Source: Blood Cancer J. 2024 Oct 31;14(1):191. doi: 10.1038/s41408-024-01178-5 (PMC11527869; doi:10.1038/s41408-024-01178-5)
Supplement: Supplementary file 1 — Supplementary data [file 41408_2024_1178_MOESM1_ESM.docx]

**Venous Thromboembolism in Adolescents and Young Adults with Acute Lymphoblastic Leukemia Treated on a Pediatric-Inspired Regimen**

Shai Shimony^1,2#^, Hari S. Raman^1#^, Yael Flamand^3^, Julia Keating^3^, Jonathan D. Paolino^4^, Yannis K. Valtis^5^, Andrew E. Place^4^, Lewis B. Silverman^4^, Stephen E. Sallan^4^, Lynda M. Vrooman^4^, Andrew M. Brunner^6^, Donna Neuberg^3^, Ilene Galinsky^1^, Jacqueline S. Garcia^1^, Eric S. Winer^1^, Martha Wadleigh^1^, Richard M. Stone^1^, Jean M. Connors^7^, Daniel J. DeAngelo^1^, Marlise R. Luskin^1^

1. Department of Medical Oncology, Dana-Farber Cancer Institute, Boston, MA, USA
2. Rabin Medical Center and Faculty of Medicine, Tel Aviv University, Israel
3. Department of Data Science, Dana Farber Cancer Institute, Boston, MA, USA
4. Department of Pediatric Oncology, Dana Farber Cancer Institute & Boston Children’s Hospital, Boston, MA, USA
5. Memorial Sloan Kettering Cancer Institute, New York, NY, USA
6. Leukemia Program, Massachusetts General Hospital, Boston, MA, USA
7. Brigham and Women's Hospital and Harvard Medical School, Boston, MA, USA

**Corresponding author:**

Shai Shimony, MD

Dana-Farber Cancer Institute

450 Brookline Ave., Mayer 1B25

Boston, MA 02215

(857) 215-0340

Shai_shimony@dfci.harvard.edu

**Supplemental Data**

Tables: 6

Figures: 4

|  | | **Hyperdiploid** | | |
| --- | --- | --- | --- | --- |
|  | | **No (N=307)** | **Yes (N=34)** | **P-value** |
| **Age, continuous (median, IQR)** | | 24.3  (17.8, 34.1) | 17.0  (16.2, 18.7) | <0.0001 |
| **BMI** | **Normal/Underweight** | 166 (54.1) | 27 (79.4) | 0.0056 |
|  | **Overweight/Obese** | 141 (45.9) | 7 (20.6) |  |

**Supplemental Table 1.** Association between Hyperdiploid karyotype, age and BMI.

IQR – interquartile range. BMI – body mass index.

|  | **Patients** | | | | | | | | | |
| --- | --- | --- | --- | --- | --- | --- | --- | --- | --- | --- |
|  | **1** | **2** | **3** | **4** | **5** | **6** | **7** | **8** | **9** | **10** |
| **CNS VTE Date** | 12/28/2003 | 1/13/2007 | 4/24/2007 | 3/26/2008 | 7/3/2008 | 3/25/2009 | 8/3/2009 | 5/26/2010 | 9/20/2019 | 6/5/2018 |
| **Phase of Treatment** | Consolidation | Induction | Consolidation | Consolidation | Consolidation | Consolidation | Consolidation | Induction | Induction | Induction |
| **VTE Prophylaxis** | No | No | N/A | No | N/A | N/A | No | No | Yes | Yes |
| **Re-challenge with ASP** | Yes (5 additional weeks for 8 weeks in total) | No (received a single dose during induction) | Yes (26 weeks documented) | Yes (30 weeks documented) | No (10 weeks in total documented) | Unknown | Yes (30 weeks documented) | Yes (30 weeks documented) | No (received a single dose during induction) | No (received a single dose during induction) |
| **Neurologic Sequelae** | - R sided weakness, aphasia, and confusion | - Altered mental status, seizures requiring intubation, and Neuro ICU admission - Symptoms resolved within 11 days with no further CNS bleeding on protocol | N/A | - Confusion and somnolence | N/A | N/A | - Headaches | - Altered mental status and seizures requiring Neuro ICU admission | - RUE weakness and word finding difficulties requiring extensive rehab - Continues to have mild executive function deficits | - R hemiplegia requiring 28 days of rehab, subsequently resolved to baseline |
| **Additional Considerations** | - Died of meningitis 6 months following CNS event | - Per documentation, considered as 2 separate CNS VTE events given evidence of R vertebral artery clot and cerebral sinus bleeding on same day - Died on 5/26/2007 after relapsing and developing DIC while on clofarabine with multifocal bleeding and CNS herniation | N/A | - No further CNS events and no lasting neurological deficits | N/A | N/A | N/A | - No further CNS events and no lasting neurological deficits | - Underwent AlloHSCT with no further CNS events | - Underwent AlloHSCT and had late extramedullary relapse, no further CNS events |

**Supplemental Table 2. Trajectory of patients with cerebral sinus vein thrombosis (CSVT)**. CNS: central venous system; VTE: venous thromboembolism; N/A: not available; ASP: asparaginase; ICU: intensive care unit; RUE: right upper extremity; DIC – disseminated intra-vascular coagulopathy; AlloHSCT: allogeneic hematopoietic bone marrow transplantation.

|  | **HR (95% CI)** | **p-value** |
| --- | --- | --- |
| Age (years) | 1.00 (0.98-1.02) | 0.72 |
| Sex (Female vs. Male) | 1.09 (0.75-1.60) | 0.65 |
| BMI Category  (Overweight/Obese vs. Normal/Underweight) | 1.45 (1.01-2.09) | 0.047 |
| WBC at diagnosis | 1.0 (0.998-1.0) | 0.89 |
| CNS (CNS-3 *vs.* CNS-1 or CNS-2) | 1.04 (0.45-2.41) | 0.93 |
| Immunophenotype (T-ALL vs. B-ALL) | 1.34 (0.91-1.97) | 0.14 |
| PEG-Asparaginase (PEG vs. no PEG) | 1.20 (0.82-1.74) | 0.35 |
| PPx Amendment (Post vs. Pre)* | 1.06 (0.71-1.58) | 0.77 |

**Supplemental Table 3**. **Univariate competing risk model for venous thromboembolism (VTE) in the Consortium cohort.** No multivariable model could be built, as no variables remained significant (at the p<0.1 level) in the multivariable setting other than BMI. HR: hazard ratio; CI: confidence interval; BMI: body mass index; WBC: white blood cells; CNS: central nervous system; ALL – acute lymphoblastic leukemia; PEG – pegylated; PPx – prophylaxis. * At 09/2011, after finding high prevalence of VTE events in the 06-254 protocol, any patients on the 06-254 or treated as per 06-254 received thromboprophylaxis during asparaginase treatment and antithrombin repletion when < 30%.

|  | **Univariate HR [95% CI]** | **p-value** | **Multivariable HR  [95% CI]** | **p-value** |
| --- | --- | --- | --- | --- |
| Thrombosis (as time-varying covariate) | 1.19 [0.62-2.30] | 0.60 | 1.45 [0.74-2.84] | 0.29 |
| Age as continuous variable (years) | 1.01 [0.97-1.04] | 0.69 | - | - |
| Sex (Male *vs.* Female) | 0.97 [.49-1.89] | 0.92 | - | - |
| BMI  (overweight/obese *vs.* normal/underweight) | 1.13 [0.59-2.19] | 0.71 | - | - |
| WBC at diagnosis (as continuous variable) | 1.002 [1.001-1.003] | <0.0001 | 1.002 [1.001-1.003] | <0.0001 |
| CNS-3 *vs.* CNS-1 or CNS-2 | 1.45 [0.20-10.61] | 0.71 | - | - |
| Immunophenotype B- *vs.* T-ALL | 5.04 [1.55-16.45] | 0.007 | 5.16 [1.57-16.93] | 0.007 |
| Hyperdiploid (yes *vs.* no) | 1.08 [.42-2.79] | 0.87 | - | - |
| MLL rearrangement (yes *vs.* no) | 1.74 [0.42-7.26] | 0.45 | - | - |
| Asparaginase completion (yes *vs.* no) | 0.65 [0.32-1.36] | 0.25 | - | - |
| Weeks of Asparaginase (as continuous variable) | 0.98 [0.95-1.02] | 0.31 | - | - |

**Supplemental Table 4. Univariate and multivariable event-free survival Cox regression landmark analysis for patients on treatment for at least one year.**

HR: hazard ratio; CI: confidence interval; BMI: body mass index; WBC: white blood cells; CNS: central nervous system; ALL: acute leukemia lymphoma; MLL: mixed lineage leukemia.

|  | **VTE group** | | | | | |  |
| --- | --- | --- | --- | --- | --- | --- | --- |
|  | **No**  **(N=122)** | | **Yes**  **(N=69)** | | **Overall**  **(N-191)** | | **P-value*** |
|  | **N** | **(%)** | **N** | **(%)** | **N** | **(%)** |  |
| **Sex (Male)** | 72 | (59.0) | 42 | (60.9) | 114 | (59.7) | 0.80 |
| **Age, continuous (median, IQR)** | 25.6 (18.8, 33.5) | | 23.7 (20.4, 35.6) | | 25.1 (19.3, 34.1) | | 0.80 |
| **Age** |  | | | | | | 0.89 |
| **15-29 years** | 79 | (64.8) | 44 | (63.8) | 123 | (64.4) |  |
| **30-50 years** | 43 | (35.2) | 25 | (36.2) | 68 | (35.6) |  |
| **BMI group (per CDC guidelines)^1^** |  | | | | | | 0.03 |
| **Normal/Underweight** | 69 | (56.6) | 28 | (40.6) | 97 | (50.8) |  |
| **Overweight/Obese** | 53 | (43.4) | 41 | (59.4) | 94 | (49.2) |  |
| **WBC at diagnosis (x10^9^/L, median, IQR)** | 16.2 (4.6, 73.0) | | 11.3 (4.9, 51.1) | | 14.8 (4.6, 64.0) | | 0.48 |
| **Fibrinogen at diagnosis (mg/dL, median, IQR)** | 359.0 (279.0, 418.0) | | 383.0  (298.0, 489.0) | | 364.5  (281.5, 449.5) | | 0.07 |
| **Immunophenotype** |  | | | | | | 0.09 |
| **B-ALL** | 84 | (68.9) | 39 | (56.5) | 123 | (64.4) |  |
| **T-ALL** | 38 | (31.1) | 30 | (43.5) | 68 | (35.6) |  |
| **CNS involvement^2^** |  | | | | | | 0.69 |
| **CNS-1** | 98 | (80.3) | 51 | (73.9) | 149 | (78.0) |  |
| **CNS-2** | 13 | (10.7) | 10 | (14.5) | 23 | (12.0) |  |
| **CNS-3** | 2 | (1.6) | 2 | (2.9) | 4 | (2.1) |  |
| **Anterior mediastinal mass^3^** | 32 | (26.2) | 24 | (34.8) | 56 | (29.3) | 0.20 |
| **Cytogenetics** |  | | | | | |  |
| **Normal** | 34 | (27.9) | 20 | (29.0) | 54 | (28.3) | 0.87 |
| **Complex** | 5 | (4.1) | 1 | (1.4) | 6 | (3.1) | 0.42 |
| **Hyperdiploid** | 12 | (9.8) | 2 | (2.9) | 14 | (7.3) | 0.09 |
| **KMT2A rearrangements (MLL)** | 15 | (12.3) | 4 | (5.8) | 19 | (9.9) | 0.21 |
| **Treatment type (On trial vs. per protocol)** | 76 | (62.3) | 35 | (50.7) | 111 | (58.1) | 0.12 |
| **Asparaginase type^4^ (PEG vs. non-PEG)** | 76 | (62.3) | 44 | (63.8) | 120 | (62.8) | 0.80 |
| **VTE PPx era^5^ (vs. pre-PPx era)** | 70 | (57.4) | 39 | (56.5) | 109 | (57.1) | 0.91 |
| **AlloHSCT** | 28 | (23.0) | 10 | (14.5) | 38 | (19.9) | 0.16 |

**Supplemental Table 5. DFCI/HCC patients (n=191) characteristics.**

^1^BMI Cutoff: For patients ≥20 years, normal (including underweight): <24.99, overweight/obese: >30. For patients 15-19, age-adjusted percentiles per CDC were used where overweight/obese ≥85%; ^2^Fifteen patients had traumatic tap/unknown CNS status; ^3^Two patients mediastinal mass evaluation was unknown; ^4^One patient type of asparaginase was unknown; ^5^At 09/2011, after finding high prevalence of VTE events in the 06-254 protocol, any patients on the 06-254 or treated as per 06-254 received thromboprophylaxis during asparaginase treatment and antithrombin repletion when <30%. VTE: venous thromboembolism; IQR: interquartile range; BMI: body mass index; CDC: Centers for Disease Control; WBC: white blood cell count; ALL: acute lymphoblastic leukemia; CNS: central nervous system; MLL: mixed-lineage leukemia; PEG: pegylated; PPx: prophylaxis; AlloHSCT: allogeneic hematopoietic stem cell transplantation.

|  | **HR (95% CI)** | **p-value** |
| --- | --- | --- |
| Age (years) | 1.01 (0.98-1.03) | 0.66 |
| Sex (Female vs. Male) | 0.98 (0.60-1.59) | 0.93 |
| Fibrinogen at diagnosis (<150 vs. ≥150 mg/dL) | 2.67 (0.84, 8.48) | 0.097 |
| BMI Category  (Overweight/Obese vs. Normal/Underweight) | 1.79 (1.10-2.89) | 0.018 |
| WBC at diagnosis | 1.0 (0.998-1.002) | 0.73 |
| CNS (CNS-3 *vs.* CNS-1 or CNS-2) | 1.13 (0.28-4.60) | 0.87 |
| Immunophenotype (T-ALL vs. ALL) | 1.62 (1.005-2.61) | 0.048 |
| PEG-Asparaginase (PEG vs. no PEG) | 1.01 (0.62-1.67) | 0.96 |
| PPx (Yes vs. No, time-varying) | 0.98 (0.56-1.72) | 0.96 |

**Supplemental Table 6. Univariate Cox proportional hazards regression model for venous thromboembolism (VTE)-free time at the DF/HCC cohort.** After the process of backwards selection for the building of the multivariable model, no variables remained significant (at the p<0.05 level) in the multivariable setting other than BMI. HR: hazard ratio; CI: confidence interval; BMI: body mass index; WBC: white blood cells; CNS: central nervous system; ALL: acute lymphoblastic leukemia; PEG: pegylated; PPx: prophylaxis


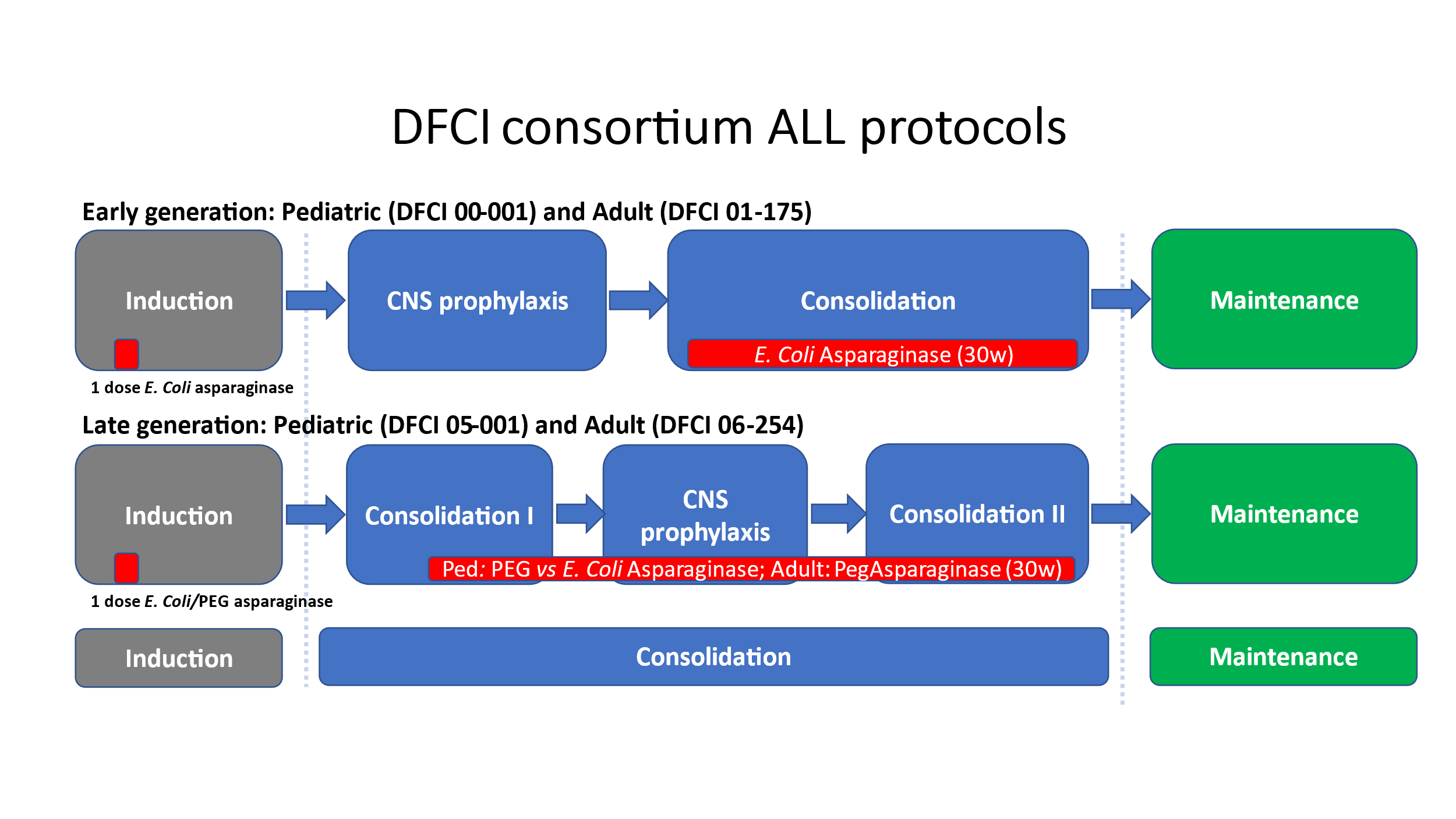


**Supplemental Figure 1. DFCI consortium protocols.**

DFCI: Dana-Farber Cancer Institute; CNS: central nervous system; PEG; pegylated. *At 09/2011, after finding high prevalence of VTE events in the 06-254 protocol, any patients on the 06-254 or treated as per 06-254 received thromboprophylaxis during asparaginase treatment and antithrombin repletion when <30%.


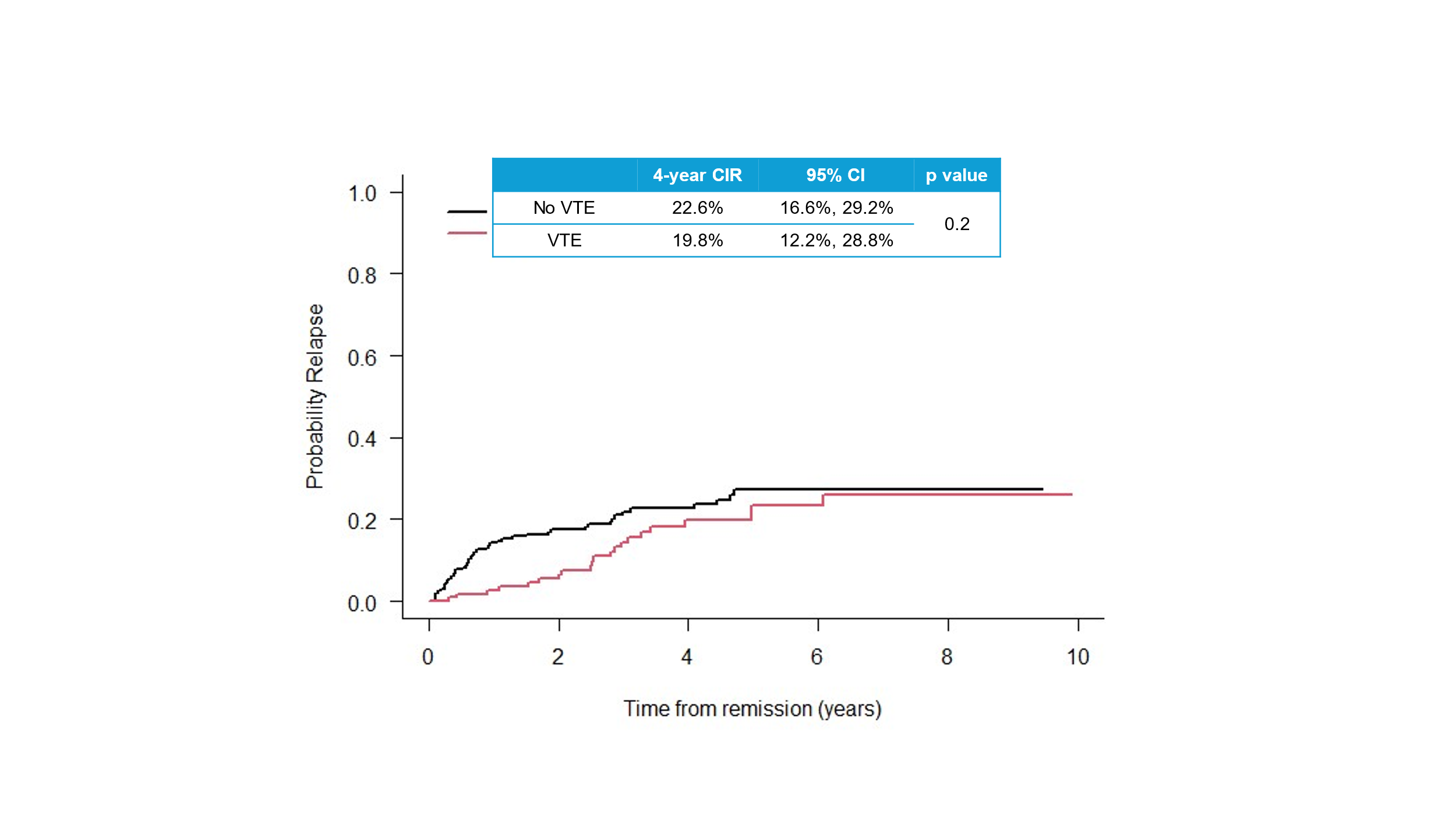


**Supplemental Figure 2. Cumulative incidence of relapse in patients with vs. without venous thromboembolism (VTE).** CIR: cumulative incidence of relapse; CI: confidence interval; VTE: venous thromboembolism.


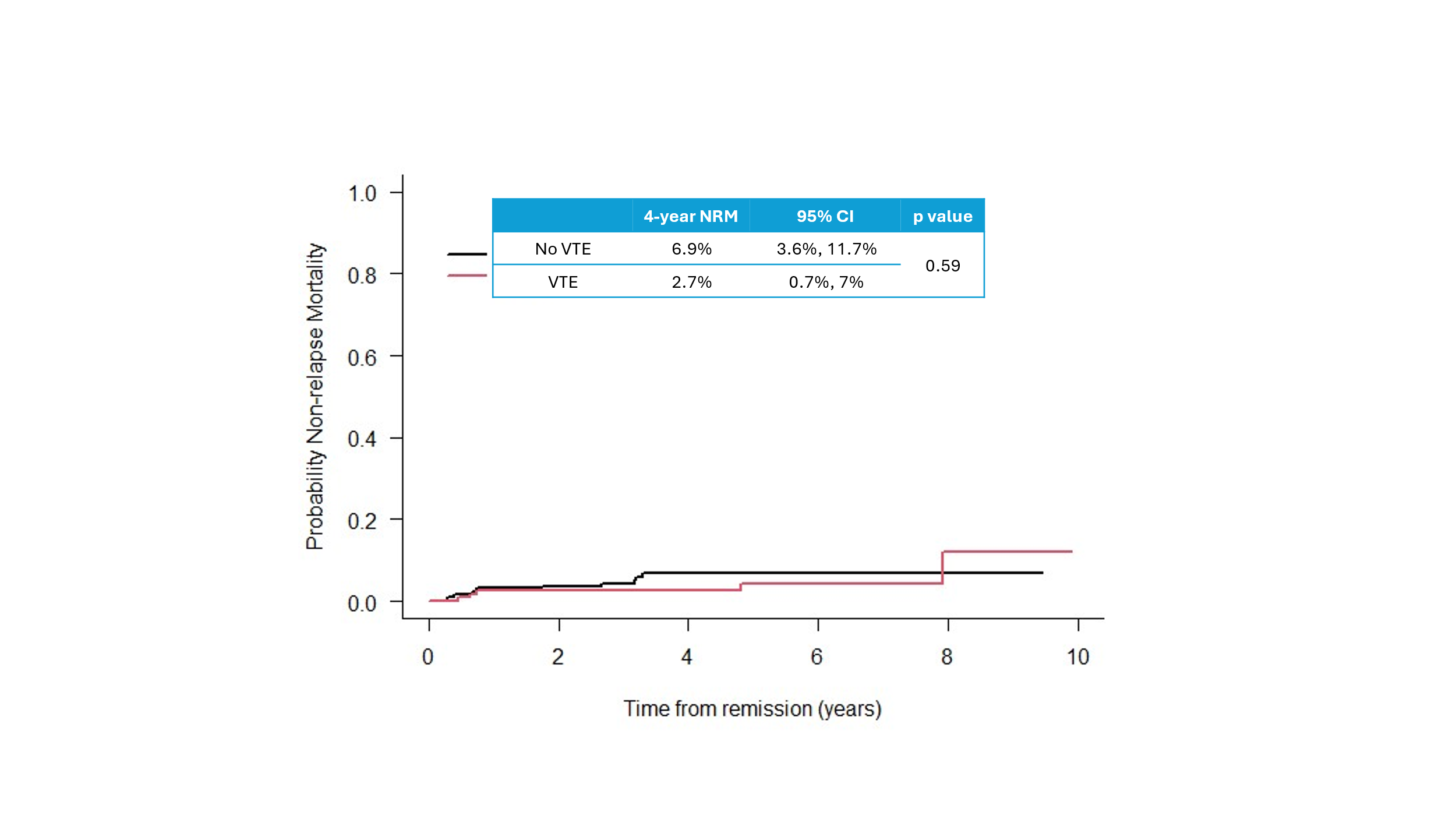


**Supplemental Figure 3. Cumulative incidence of non-relapse mortality in patients with vs. without venous thromboembolism (VTE).** NRM: non-relapse mortality; CI: confidence interval; VTE: venous thromboembolism.


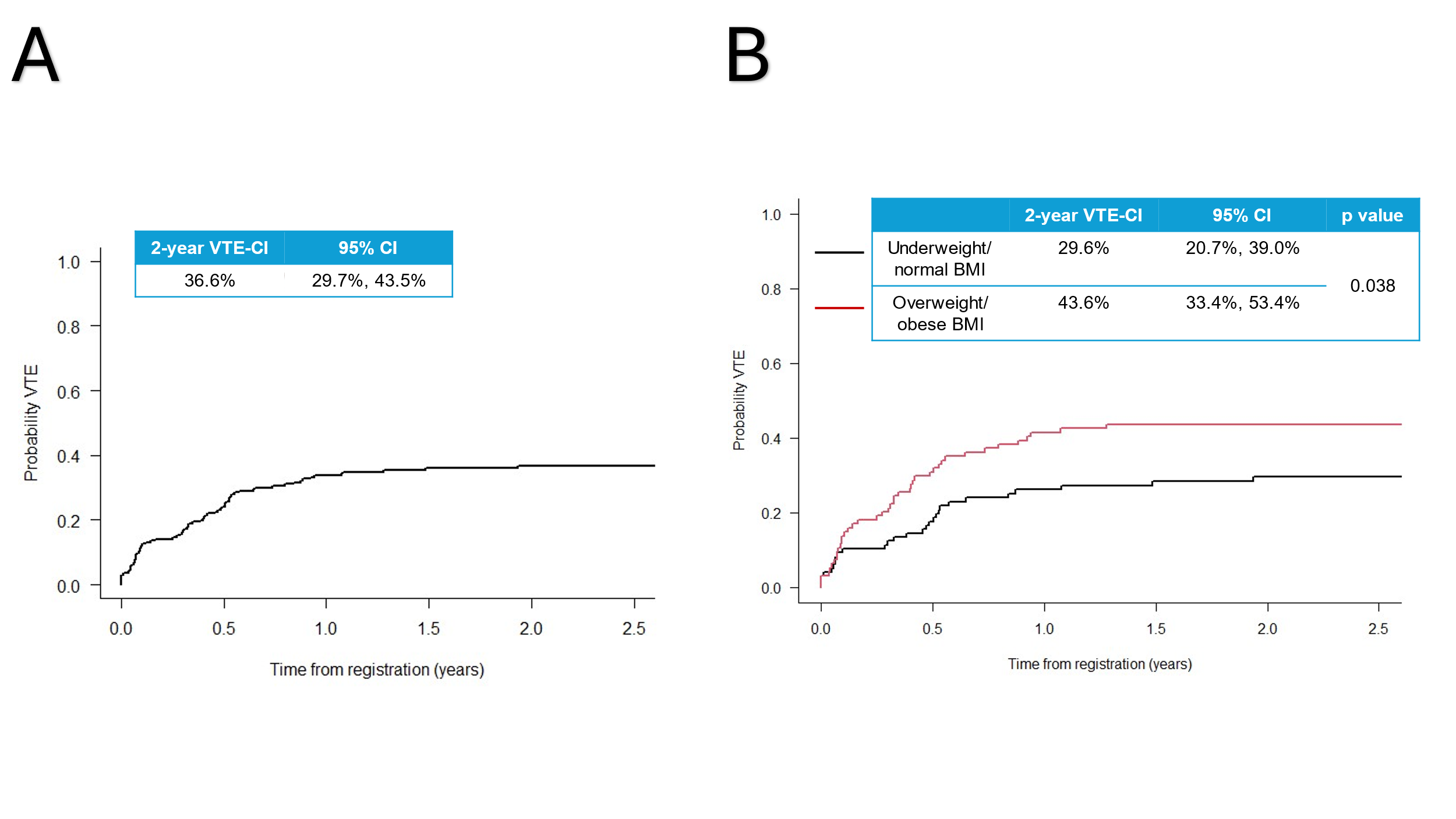


**Supplemental Figure 4. 2-year cumulative incidence of venous thromboembolism (VTE in the DFCI/HCC cohort (n=191).** A. All DFCI/HCC cohort; B. Stratified by BMI groups. VTE-CI: venous thromboembolism cumulative incidence; CI: confidence interval; BMI: body mass index.
